# Adapting NeuroVanguard to real-world challenges

**DOI:** 10.1186/s13054-024-04916-0

**Published:** 2024-04-23

**Authors:** Andres Giglio, Monserrat Pino, Andres Ferre, Andres Reccius

**Affiliations:** 1 Critical Care Department, Finis Terrae University, Santiago, Chile; 2 Critical Care Department, Clinica Las Condes Hospital, Santiago, Chile; 3 Critical Care Program Director, Finis Terrae University, Santiago, Chile; 4 Neurocritical Care Program Director, Finis Terrae University, Santiago, Chile; 5 Neurology Department, Clinica Las Condes Hospital, Santiago, Chile

Dear Editor,

We read with great interest the review by Rodriguez et al. on the "NeuroVanguard" strategy for neuromonitoring in severe adult brain injury patients [1]. The authors present a compelling case for combining noninvasive and invasive monitoring tools to enhance treatment customization and improve care quality. However, we believe that certain aspects of this approach warrant further consideration when faced with real-world challenges.

Firstly, the authors acknowledge the limited access to advanced neuromonitoring tools in resource-constrained settings. Intracranial pressure (ICP) monitoring, brain tissue oxygenation (PbtO2) assessment, and cerebral microdialysis remain scarce in most healthcare facilities worldwide, being not a universal standard of care but the optimal gold standard in settings with maximum resource availability.

In real-world resource-constrained scenarios, we agree with the authors' suggestion of using readily available clinical assessments and systemic physiological monitoring, such as the "GHOST-CAP" variables [1]. These systematized evaluations are part of the daily routine in the care of critically ill patients. In the complex and challenging context of neurocritical care, it is essential to perform these ordinary tasks extraordinarily well to achieve the desired outcomes. Prompt recognition and correction of systemic derangements have been shown to improve outcomes in brain-injured patients [2].

Secondly, we strongly believe that the most valuable and complex neuromonitoring tool is the repeated and systematic neurological examination. This examination should not be limited to neurologists or intensivists. Neurocritical care trained nurses, who spend the most time with patients, play a crucial role in detecting and mitigating secondary injuries [3]. Empowering and training these nurses in all available assessment tools should be a top priority.

When faced with an unexaminable patient in a high-resource setting, multimodal neuromonitoring should be considered the second-best option. In such cases, relying on individual monitoring tools may not provide a comprehensive understanding of the brain's condition. Instead, we advocate for a combination of invasive and noninvasive approaches that aim to simulate the complexity of the neurocritical examination. By integrating data from various monitoring modalities, clinicians can gain a more holistic view of the patient's cerebral status and guide targeted interventions [4].

Furthermore, we would like to underscore the valuable approach mentioned in the paper, which highlights that absolute and isolated values lack the same relevance as considering the evolution, trajectories, and combination of values obtained from multimodal neuromonitoring. This perspective is particularly important when assessing ICP and cerebral perfusion pressure (CPP) in patients with severe brain injury. Rather than focusing solely on isolated ICP and CPP values, clinicians should consider the trends and patterns of these parameters in conjunction with other neuromonitoring modalities, such as brain tissue oxygenation, cerebral blood flow, and metabolic markers. This comprehensive approach can provide a more accurate assessment of the patient's cerebral status and guide targeted interventions.

While elevated ICP is a significant concern, focusing solely on a normal ICP, as shown in the algorithm, may overlook patients suffering from systemic low flow or oxygenation with cerebral impact. As the main goal of neurocritical care is to maintain adequate cerebral blood flow and oxygenation, a more comprehensive evaluation, as outlined in the SIBICC consensus (2020), is essential [5]. This can include the use of PbtO2 monitoring and/or near-infrared spectroscopy (NIRS) to assess cerebral oxygenation. The integration of these monitoring techniques with ICP and CPP monitoring can provide a more comprehensive assessment of cerebral perfusion and guide targeted interventions to optimize brain oxygenation and minimize secondary brain injury.

In the NeuroVanguard approach, the second step focuses on brain compliance. However, we believe that cerebral autoregulation, rather than compliance, is the key element in defining the risk of neurological damage after systemic injury. Cerebral autoregulation refers to the brain's ability to maintain relatively constant cerebral blood flow despite changes in CPP. Impaired cerebral autoregulation has been associated with worse outcomes in patients with severe brain injury. By identifying the brain's capability to adapt to neurocritical changes, clinicians can better understand the risk of secondary brain injury and guide potential therapies to improve clinical outcomes. Not only relevant as part of the risk diagnosis, knowing the autoregulatory status defines the availability of its use for therapeutic measurements, such as MAP trials described in the SIBICC consensus [5].

In conclusion, while we commend the authors for their detailed review of the NeuroVanguard approach, its real-world applicability may be limited by resource constraints and an overemphasis on technology. Prioritizing readily available clinical assessments, empowering neurocritical care nurses, and adopting a holistic view of cerebral perfusion are crucial for optimizing outcomes in severe brain injury patients.

## Data Availability

Not applicable. All cited articles are available in the respective journals.

## Abbreviations

ICP: Intracranial pressure
PbtO2: Brain tissue oxygenation
CPP: Cerebral perfusion pressure
SIBICC: Seattle International Severe Traumatic Brain Injury Consensus Conference
NIRS: Near-infrared spectroscopy
MAP: Mean arterial pressure
